# Common Commensal Cancer Viruses

**DOI:** 10.1371/journal.ppat.1006078

**Published:** 2017-01-19

**Authors:** Patrick S. Moore, Yuan Chang

**Affiliations:** University of Pittsburgh Cancer Institute, Pittsburgh, Pennsylvania, United States of America; University of Florida, UNITED STATES

The human microbiome—more properly called the bacteriome—has moved from the backwaters of microbiology to the forefront of clinical science and opened new approaches to diseases, including autoimmune disorders, cancer, and malnutrition. Human virome analyses, however, are at a nascent stage and disadvantaged by the necessity for detecting viruses (without highly conserved consensus sequences such as the ribosomal RNA genes) in the background of the abundant human genome. Overcoming this hurdle generally requires additional manipulations, such as nuclease protected sequencing [1,2], rolling circle amplification [3], or digital transcriptome subtraction [4]. Nonetheless, it is clear that we possess a rich and diverse virome that probably contributes to our health but also, when perturbed, causes diseases, including cancers.

## Cancer, Viruses, and Causality

Cancer viruses give us an important view on causation theory due to the peculiarities of their biology. Unlike acute viral infections, in which common sense and simple epidemiology provide an answer as to whether or not an agent causes a disease, viral cancers have complex patterns of infection that reveal fundamental weaknesses in current epidemiologic theory [5,6].

Tumor formation is not an evolutionary adaptation for any human tumor virus discovered thus far, and when viral tumors occur, they are biological accidents. There are seven established human cancer viruses (HIV is also considered a cancer virus, but it appears to cause tumors through its immunosuppressive effects, and BK polyomavirus is emerging as a likely cause of some transplant-related urinary cancers[7]), but none of these agents cause cancer in the majority of infected persons—cancers are not a fundamental part of these viruses’ life cycles [8]. Instead, cancers are evolutionarily dead-end events that threaten the viral agent as much as the host, and viral tumors only occur in a minor fraction of infected individuals when multiple factors exist together with infection, such as specific gene mutations or immune system suppression. Furthermore, viruses in these tumors are generally nonpermissive for forming infectious particles—or only marginally able to replicate—and so tumor-based transmission of virus infection within the human population is virtually nil. Most virus transmission, which is the fulcrum for viral evolution, occurs between asymptomatic persons. Virtually all viral cancers are highly increased in immunosuppressed persons (e.g., post-transplant and AIDS patients), consistent with continued immune system surveillance being critical in controlling outgrowth of a tumor once someone is infected [9,10].

## Direct and Indirect Carcinogens

Tumor viruses have been divided into direct carcinogens, in which intracellular viral oncogenic antigens drive cell proliferation, and indirect carcinogens, in which virus-induced chronic inflammation initiates cancer cell growth rather than a viral oncogene [11].

For direct viral carcinogens, the interplay between immune system surveillance and infection can be readily explained: expression of a viral oncoprotein in each cancer cell provides a foreign antigen that can be recognized by an intact immune system. Kaposi sarcoma (KS), for example, caused by Kaposi sarcoma herpesvirus (KSHV), is increased tens-of-thousands–fold among AIDS and transplant patients compared to the general populations from which these patients are drawn [9]. A role for immune surveillance is surprisingly true even for gastric carcinoma, in which a hit-and-run mechanism for *Helicobacter pylori* can be expected. While Epstein-Barr Virus (EBV) has been implicated in a subset of gastric carcinoma, *H*. *pylori* is likely to act as a direct carcinogen, similar to tumor viruses. Studies have shown that Type IV intercellular transport of bacterial CagA oncoprotein from bacterium to host cells may be analogous to a direct carcinogenesis model for viruses, and might stimulate an immune surveillance response [12].

For tumor virus discovery, the relationship to immunity has been particularly useful. Merkel cell polyomavirus (MCV), the cause for most Merkel cell carcinomas (MCC), was only sought [13] because MCC rates are elevated among AIDS patients [14]. Unsurprisingly, immune checkpoint inhibitors appear to be potent single agents in achieving durable remission for some viral cancers compared to nonviral cancers [15]. Although an immune dependence for the development of viral tumors is very strong, this should not be considered absolute since a virus that expresses only oncogenic miRNAs or long noncoding RNAs might escape immune surveillance.

An indirect mechanism of carcinogenesis has always been an intriguing possibility in explaining the role of many viruses that have been accused of being cancer culprits (the so-called hit-and-runners). But instead, hepatitis C virus (HCV) in hepatocellular carcinoma may be the only documented example of true hit-and-run, although most HCV-induced hepatocellular carcinomas retain some level of HCV infection and HCV Nonstructural protein 5 is tumorigenic in transgenic mice.

## Pathogenic Versus Commensal Tumor Viruses

Another way to look at infectious carcinogens is whether or not a tumor virus is a rare or a common human infection. Commensal viruses are common, inapparent infections that do not usually cause symptoms or disease in the host. While many commensal viruses, such as EBV and MCV, were discovered because of the rare diseases they cause, subsequent studies revealed that these viruses are common and usually result in asymptomatic, persistent infections. This has important implications not just for patterns of tumor occurrence but also for how viruses are judged to cause cancer.

Most tumor viruses are not common infections, and therefore, patterns of tumor occurrence roughly reflect patterns of virus infection. Hepatitis viruses B (HBV) and C (HCV), human T cell leukemia virus I (HTLV I), human papillomavirus (HPV), and KSHV are generally uncommon, at least in North American and European populations, and so risk factors for infection in these populations are also risk factors for their corresponding cancers. For sexually transmitted tumor viruses, particularly KSHV, HPV, and HBV, sexual risk factors for infection (e.g., unprotected intercourse, number of sex partners) are prominent. With these viruses, population-based changes in sexual behavior can lead to marked changes in cancer risk: increased “safe-sex” behaviors may have contributed to declines since the 1980s for HBV- and KSHV-related cancer incidence, as well as the more obvious effects of effective antiretroviral therapy among HIV/AIDS patients. In contrast, increased acceptance of oral-genital sex in the United States may be contributing to a marked increase in HPV-related male head and neck cancer rate [16] (HPV is notable for not disseminating after infection and so specific sexual risk behaviors can be epidemiologically linked to site-specific cancers).

These “pathogenic” cancer viruses do not differ conceptually from other acute disease-causing viruses: exposure and infection is the primary determinant for disease, although infection alone does not cause cancer. Epidemiologists generally use Hill’s criteria to determine causality, first developed to address the question of whether or not exposure to cigarette smoking causes lung cancer [17] (Koch’s postulates—frequently cited by non-experts—were not first conceptualized by Robert Koch [18], and were made obsolete in the late 1800s by Koch’s own discovery of asymptomatic cholera infection [19] and by the discovery of viruses and obligate cell-associated agents that could not be isolated in pure culture) [20]. Hill’s criteria are applied to address questions such as, “Is the virus infection specific to the suspect cancer?” and “Does virus infection precede cancer appearance?” For KS, epidemiologists predicted that it should be caused by a novel, non-ubiquitous virus [21] prior to discovery of KSHV [22]. Blood tests were rapidly developed, and, by using cohorts already enrolled to study HIV, the epidemiologists’ predictions were fulfilled and Hill’s criteria could be rapidly and convincingly affirmed for KSHV causing KS [23].

A much more complicated situation arises, however, when viruses that are common commensals (e.g., EBV and MCV) cause cancer under certain specific circumstances. EBV is highly prevalent among adults (>95%) and so risk factors for EBV infection are generally not linked to EBV-related cancers. Similarly, MCV is a common commensal virus on our skin (prevalence ~60% among adults) therefore MCV infection is also not a strong risk factor for the MCC. Recent studies reveal that there are 12 other human polyomaviruses, and all but one live as common commensal flora in humans [24]. For some viruses, such as KSHV in sub-Saharan and Andean populations, infection is also near ubiquitous.

How can a near-universal infection like EBV cause a rare cancer like Burkitt lymphoma? This question stymied epidemiologists for 31 years after its discovery and EBV was not designated as an International Agency for Research on Cancer (IARC) carcinogen until 1996. This decision was not based on studies of the most notorious cancers caused by EBV, such as Burkitt lymphoma and nasopharyngeal carcinoma. Instead, it required studies on a rare X-linked lymphoproliferative disorder, which could be shown to be directly related to de novo EBV infection among susceptible children. Hill’s criteria largely failed to address this controversy.

Epidemiology is based on comparisons among individuals (infected versus uninfected, with cancer versus without cancer), which may not adequately assess our modern view of multifactorial disease causation. To better address commensal tumor viruses, one needs to turn to molecular biology. MCV was found to be clonally integrated into MCC tumor cells [13] and thus the virus was present in proto-tumor cells before the beginning of cancer cell clonal expansion [25]. In tumors, but not in commensal healthy tissue infections, MCV is mutated in one of its oncoproteins (large T antigen) that serves as the virus’ major replication protein, and so it is not possible for the virus to ex post facto infect pre-existing tumor cells as a passenger infection. Remarkably, MCV-driven MCC does not appear to depend on specific host cell mutations but rather on mutations to a common, extra-human floral viral component. Furthermore, simple immunostaining for large T antigen reveals that tumor cells are uniformly infected with the virus whereas surrounding healthy tissues are not and T antigen expression is required for proliferation of MCV-MCC tumor cells [25]. These findings have little weight in traditional epidemiologic assessments of causality but are fundamental to a molecular biologic understanding of cancer occurrence. MCV is the causal agent for most—but not all—MCC tumors based on the molecular evidence.

**Fig 1:**
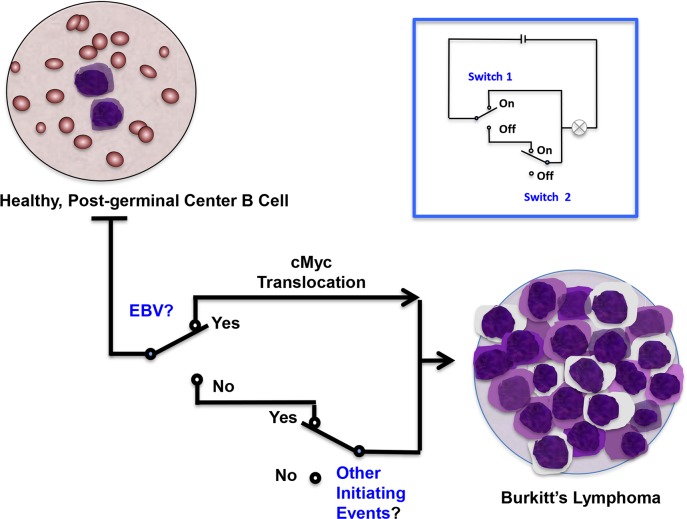
Classical epidemiology is poorly equipped to determine multifactorial causality for common commensal tumor viruses, such as EBV. Multifactorial causal reasoning is shown for a simple electrical circuit (inset) with two switches, Switch 1 and Switch 2, either of which can “cause” the light bulb to turn on. An analogous pathway is shown for the genesis of Burkitt lymphoma, in which EBV is responsible for a portion of tumors, but also only in the biological context of other factors, such as cMYC translocations. Since EBV is nearly ubiquitous, teasing out its contribution to a rare cancer like Burkitt lymphoma is supremely difficult using standard epidemiologic methods, but is readily evident using molecular biologic information that has been available for decades. EBV is clonal in these tumors based on terminal repeat copies and Epstein–Barr encoding region (EBER) in situ hybridization typically reveals the presence of EBV genome in all tumor cells but not surrounding nontumor cells. These facts are biologically implausible for a non-causal passenger infection [6].

Commensal viruses have generally evolved to avoid causing symptomatic disease and only do so in specific host settings, such as immune suppression. Determining causality requires measuring the virus infection in the context of these factors rather than infection alone. Thus, EBV infection alone does not cause cancer but EBV infection of a post-germinal B cell having a c-Myc rearrangement does. A 21st century epidemiologist should not ask if EBV infection alone is associated with a specific cancer but whether EBV in a specific molecular context is associated with disease (Fig 1).

To truly determine whether a candidate virus is a cancer agent requires effective vaccines or antivirals. If eliminating the infection also eliminates the cancer, then the evidence is clear. But, as shown by history, specific drug and vaccine development will only occur once a virus is already established to cause a disease or cancer, and there is sufficient economic interest on the part of biopharmaceutical companies. Animal models suggest some commensal virus infections are beneficial to host immune responses, and so, modeling a cost-benefit analysis may have to be considered for future vaccines that might prevent these infections [26]. The payoff for establishing a virus as a cause for cancer, however, is enormous and can be seen in the ways that HBV and HPV vaccines have changed age-old patterns of cancer.

## References

[1] Gaynor AM, Nissen MD, Whiley DM, Mackay IM, Lambert SB, et al (2007) Identification of a novel polyomavirus from patients with acute respiratory tract infections. PLoS Pathog 3: e64 10.1371/journal.ppat.0030064 17480120 PMC1864993

[2] Allander T, Andreasson K, Shawon G, Bjerkner A, Bogdanovic G, et al (2007) J Virol 81:4130–36. 17287263 10.1128/JVI.00028-07 PMC1866148

[3] Schowalter RM, Pastrana DV, Pumphrey KA, Moyer AL, Buck CB (2010) Merkel Cell Polyomavirus and Two Previously Unknown Polyomaviruses Are Chronically Shed from Human Skin. Cell Host Microbe 7: 509–515. 10.1016/j.chom.2010.05.006 20542254 PMC2919322

[4] Feng H, Taylor JL, Benos PV, Newton R, Waddell K, et al (2007) Human transcriptome subtraction by using short sequence tags to search for tumor viruses in conjunctival carcinoma. J Virol 81: 11332–11340. 10.1128/JVI.00875-07 17686852 PMC2045575

[5] Moore PS, Chang Y (1998) Kaposi's sarcoma (KS), KS-associated herpesvirus, and the criteria for causality in the age of molecular biology. Am J Epidemiol 147: 217–221. 9482495 10.1093/oxfordjournals.aje.a009440

[6] Moore PS, Chang Y (2014) The conundrum of causality in tumor virology: the cases of KSHV and MCV. Seminars in cancer biology 26: 4–12. 10.1016/j.semcancer.2013.11.001 24304907 PMC4040341

[7] Kenan DJ, Mieczkowski PA, Burger-Calderon R, Singh HK, Nickeleit V (2015) The oncogenic potential of BK-polyomavirus is linked to viral integration into the human genome. J Pathol 237: 379–389. 10.1002/path.4584 26172456 PMC5042064

[8] Moore PS, Chang Y (2010) Why do viruses cause cancer? Highlights of the first century of human tumour virology. Nat Rev Cancer 10: 878–889. 10.1038/nrc2961 21102637 PMC3718018

[9] Grulich AE, van Leeuwen MT, Falster MO, Vajdic CM (2007) Incidence of cancers in people with HIV/AIDS compared with immunosuppressed transplant recipients: a meta-analysis. Lancet 370: 59–67. 10.1016/S0140-6736(07)61050-2 17617273

[10] Schulz TF (2009) Cancer and viral infections in immunocompromised individuals. Int J Cancer 125: 1755–1763. 10.1002/ijc.24741 19588503

[11] Zur Hausen H (2009) The search for infectious causes of human cancers: where and why. Virology 392: 1–10. 10.1016/j.virol.2009.06.001 19720205

[12] Odenbreit S, Puls J, Sedlmaier B, Gerland E, Fischer W, et al (2000) Translocation of Helicobacter pylori CagA into gastric epithelial cells by type IV secretion. Science 287: 1497–1500. 10688800 10.1126/science.287.5457.1497

[13] Feng H, Shuda M, Chang Y, Moore PS (2008) Clonal integration of a polyomavirus in human Merkel cell carcinoma. Science 319: 1096–1100. 10.1126/science.1152586 18202256 PMC2740911

[14] Engels EA, Frisch M, Goedert JJ, Biggar RJ, Miller RW (2002) Merkel cell carcinoma and HIV infection. Lancet 359: 497–498. 10.1016/S0140-6736(02)07668-7 11853800

[15] Nghiem PT, Bhatia S, Lipson EJ, Kudchadkar RR, Miller NJ, et al (2016) PD-1 Blockade with Pembrolizumab in Advanced Merkel-Cell Carcinoma. N Engl J Med 374: 2542–2552. 10.1056/NEJMoa1603702 27093365 PMC4927341

[16] Beachler DC, Sugar EA, Margolick JB, Weber KM, Strickler HD, et al (2015) Risk factors for acquisition and clearance of oral human papillomavirus infection among HIV-infected and HIV-uninfected adults. Am J Epidemiol 181: 40–53. 10.1093/aje/kwu247 25480823 PMC4288119

[17] Hill AB (1965) Environment and disease: association or causation? Proc Roy Soc Med 58: 295–300. 14283879 10.1177/003591576505800503 PMC1898525

[18] Brock TD (1999) Robert Koch: A life in medicine and bacteriology Washington DC: ASM Press. 1–364 p.

[19] Koch R (1893) About the current state of the bacteriological diagnosis of cholera. Journal of Hygiene and Infectious Diseases 14: 319–338.

[20] Rivers TM (1937) Viruses and Koch's Postulates. Journal of bacteriology 33: 1–12. 16559982 10.1128/jb.33.1.1-12.1937 PMC545348

[21] Beral V, Peterman TA, Berkelman RL, Jaffe HW (1990) Kaposi's sarcoma among persons with AIDS: a sexually transmitted infection? Lancet 335: 123–128. 1967430 10.1016/0140-6736(90)90001-l

[22] Chang Y, Cesarman E, Pessin MS, Lee F, Culpepper J, et al (1994) Identification of herpesvirus-like DNA sequences in AIDS-associated Kaposi's sarcoma. Science 265: 1865–1869. 10.1126/science.7997879 7997879

[23] Sarid R, Olsen SJ, Moore PS (1999) Kaposi's sarcoma-associated herpesvirus: Epidemiology, virology, and molecular biology. Advances in Virus Research, Vol 52 52: 139–232. 10384236 10.1016/s0065-3527(08)60299-7

[24] DeCaprio JA, Garcea RL (2013) A cornucopia of human polyomaviruses. Nature reviews Microbiology 11: 264–276. 10.1038/nrmicro2992 23474680 PMC3928796

[25] Chang Y, Moore PS (2012) Merkel cell carcinoma: a virus-induced human cancer. Annual review of pathology 7: 123–144. 10.1146/annurev-pathol-011110-130227 21942528 PMC3732449

[26] Barton E. S., White D. W., Cathelyn J. S., Brett-McClellan K. A., Engle M., Diamond M. S., Miller V. L., and Virgin H. W. (2007). Herpesvirus latency confers symbiotic protection from bacterial infection. Nature 447**:** 326–329. 10.1038/nature05762 17507983

